# Using epigenetic clocks in environmental epigenetics: recommendations for estimating biological aging in perinatal and pediatric samples

**DOI:** 10.1042/EBC20253013

**Published:** 2025-08-04

**Authors:** Gillian England-Mason

**Affiliations:** 1 Department of Pediatrics, Cumming School of Medicine, University of Calgary, Calgary, Alberta, Canada; 2 Owerko Centre, Alberta Children’s Hospital Research Institute, University of Calgary, Calgary, Alberta, Canada

**Keywords:** aging, biomarkers, DNA methylation, early life, environmental epigenetics, environmental exposures, epigenetics

## Abstract

Following a variety of early environmental experiences and exposures, epigenetic modifications such as DNA methylation are proposed as candidate mechanisms that contribute to health and disease across the lifespan. Epigenetic clocks are a type of aging biomarker that can offer insight into age-related changes associated with early environmental exposures. This review provides a brief overview of epigenetic clocks that are readily available for use with perinatal and/or pediatric samples, as well as highlights some recent research that has studied the associations between early environmental chemical exposures and epigenetic aging rates. Broadly, the easily accessible epigenetic clocks can be categorized as chronological age estimators and gestational age estimators, but some clocks were developed for use with specific tissues and/or age groups. Previous environmental epidemiology studies have shown that early environmental exposures such as air pollutants and endocrine-disrupting chemicals are associated with altered epigenetic aging rates in perinatal and pediatric populations. However, more research is needed that examines how factors such as exposure level, timing of exposure, and sex may affect the direction and magnitude of associations. This review concludes with some recommendations and future directions for the use of epigenetic clocks in environmental epigenetics. Overall, epigenetic clocks are promising, non-causal biomarkers of early exposures that can be examined in relation to environmental chemicals, health and disease outcomes, and as biological mediators. Future research could help determine whether these clocks hold promise as informative biomarkers that reflect developmental epigenotoxicity following early exposure to environmental chemicals.

## Introduction

Epigenetic modifications are proposed as candidate mechanisms that contribute to disease risk following a variety of early experiences and exposures [1], including modifiable environmental exposures such as pollutants and toxic chemicals [2]. Epigenetic marks such as DNA methylation (DNAm), histone modification, and RNA-mediated processes represent potentially reversible mechanisms underlying disease risk and could be used to better understand developmental and age-related molecular responses to environmental factors [3]. One epigenetic biomarker that offers insight into age-related changes associated with early environmental experiences and exposures is the epigenetic clock. This mini-review provides an overview of epigenetic clocks that are easily accessible and reproducible for use with perinatal and/or pediatric tissues, as well as highlights some recent research that has examined the associations between early environmental chemical exposures and epigenetic aging rates. It will conclude with a discussion of the considerations and future directions for environmental epigenetics research that uses epigenetic clocks in perinatal and pediatric samples.

### Biological aging in perinatal and pediatric samples

Various biological hallmarks of aging and longevity have been documented, including telomere length, mitochondrial function, DNA damage, and changes in epigenetic regulation [4]. With increasing evidence of DNAm changes in age-associated diseases (e.g. cancer and neurodegeneration), measurement of DNAm patterns has emerged as an approach to estimate biological aging in human tissues [5]. Epigenetic clocks are a biomarker of aging devised using a supervised machine learning method, primarily elastic net penalized regression^1^, whereby chronological age is regressed onto methylation sites (i.e. CpGs, which are sites where a cytosine is followed by guanine and joined by a phosphate). The penalized regression model automatically selects a subset of highly age-related CpGs, and their weighted average from the regression coefficients comprises the epigenetic clock [6]. DNAm data from high-throughput technologies, such as the Illumina methylation arrays (e.g. Illumina Infinium Human Methylation450 and MethylationEPIC)^2^, have been used to create a number of epigenetic clocks that estimate DNAm-based aging in human tissues.

**Figure 1:**
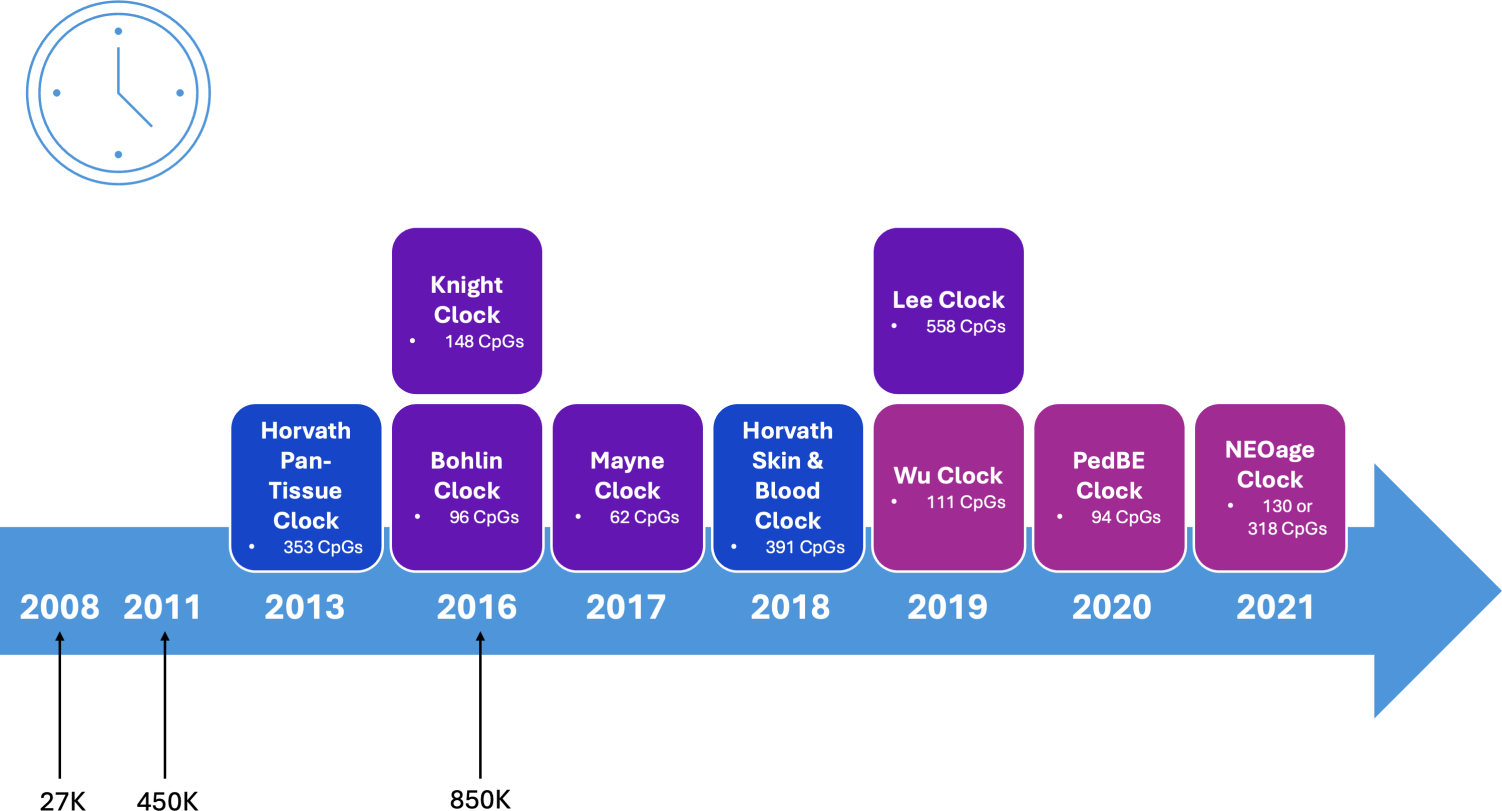
The epigenetic clocks readily available for use with perinatal and pediatric samples can be categorized as follows: (blue) epigenetic clocks developed for chronological age prediction for use across multiple tissues and the life course, (pink) epigenetic clocks developed for chronological age prediction for use with specific tissues and/or age groups, and (purple) epigenetic clocks developed for gestational age prediction for use with specific tissues and/or age groups.

An overview is provided below of epigenetic clocks that are easily accessible and have been previously used in perinatal and/or pediatric samples (Table 1). Specifically, these are epigenetic clocks that have been manualized (i.e. their estimation can be completed using publicly available code or reference values). Given the increasing interest in biological aging estimators, this may overlook epigenetic clocks that are not widely used (e.g. the epigenetic clock based on differentially methylated regions correlated with gestational age [15]). The epigenetic clocks readily available for use with perinatal and pediatric samples can be categorized as follows: (i) those developed for chronological age prediction for use across multiple tissues and the life course, (ii) those developed for chronological age prediction for use with specific tissues and/or age groups, and (iii) those developed for gestational age prediction for use with specific tissues and/or age groups (Figure 1).

**Table 1:** Summary of biological age and gestational age epigenetic clocks

Clock, year published	Tissue type(s)	Age group	Sample size (training/ testing)	Platform(s)	Number of CpGs considered	Statistical approach	Number of CpGs selected	Correlation with age
**Chronological age estimators**								
Horvath pan-tissue, 2013 [6]	51 tissues and cell types	0–100 years	7844 (datasets 1–39 used to train/ datasets 40–71 used to test)	Illumina 27K or 450K array	21,369	Elastic net regression	353	Training r=0.97, error^1^=2.9 years; Testing r=0.96, error^1^=3.6 years
Horvath skin & blood, 2018 [7]	Fibroblasts, keratinocytes, buccal cells, endothelial cells, blood, and saliva	0^6^– 94 years	2222 (896 training/ 1326 testing)	Illumina 450K or 850K array	Specific sites present on both platforms^2^	Elastic net regression	391	-
Wu, 2019 [8]	Whole blood	9–212 months	716 (K-fold cross validation used, k=10)	Illumina 27K or 450K array	21,979	Elastic net regression	111	Training r=0.98, error^1^=5.9 months; Testing r=0.98, error^1^=6.7 months
PedBE, 2020[9]	Buccal cells	0–20 years	1721 (1032 training/ 689 testing)	Illumina 450K or 850K array	-	Elastic net regression	94	Testing r=0.98, error^1^=0.35 years
NEOage, 2021 [10]	Buccal cells	Preterm infants (<30 weeks of gestation)	542 (K-fold cross validation used, k=10)	Illumina 850K array	364,410 for 450K analyses; 706,323 for 850K analyses	Elastic net regression	130 for 450K PNA 318 for 850K PNA	PNA-450K^3^ r=0.93, RMSE=1.63; PNA-850K^4^ r=0.94, RMSE=1.55
**Gestational age estimators**								
Knight, 2016 [11]	Cord blood	0 (neonates)	1342^7^ (207 training/ 1135 testing)	Illumina 27K or 450K array	16,838	Elastic net regression	148	Training r=0.99, median error=0.35 weeks; Testing r=0.91, median error=1.24 weeks
Bohlin, 2016 [12]	Cord blood	0 (neonates)	1753 (1068 training/ 685 testing)	Illumina 450K array	473,731	MM-type robust linear regression then LASSO regression	96^5^	R^2^ from 0.50 to 0.67; SE from 12.4 to 14.9
Mayne, 2017 [13]	Placenta	8–42 weeks of gestations	170 (85 training/ 85 testing)	Illumina 27K or 450K array	18,437	Elastic net regression	62	Training r=0.99, error^1^=0.23 weeks; Testing r=0.95, error^1^=1.47 weeks
Lee, 2019 [14]	Placenta	5–42 weeks of gestation	1289 (1102 training/ 187 testing)	Illumina 450K or 850K array	441,870	Elastic net regression	558	r=0.99, error^1^=0.96 weeks

1 Median absolute difference (also sometimes referred to as median ‘error’); the median absolute difference between DNA methylation-based age and chronological age.

2 Described as sites present on both platforms and those with the most significant correlations with chronological age in different cell types and least significant correlations with chronological age in different cell types.

3 NEOage Postnatal age (PNA) for the Infinium Human Methylation 450K BeadChip.

4 NEOage Postnatal age (PNA) for the Infinium MethylationEPIC BeadChip.

5 96 CpGs were selected in the LASSO models for ultrasound-based gestational age prediction using CpGs from the complete methylation data; depending on the model, for example using ultrasound-based or last menstrual period-based gestational age predictors, or which multiple testing correction method (false discovery rate or Bonferroni), 58–132 CpGs were selected.

6 Specified as -0.30 years in [8].

7 Based on Table 1 from [12].

#### Epigenetic clocks for chronological age prediction in pediatric samples

The most widely used epigenetic clock is the Horvath pan-tissue clock, which was the first multi-tissue age estimator. It determines biological age based on 353 CpGs [6]. The statistical approach (i.e. elastic net regression) used to develop this clock has since been replicated to create numerous age predictors using omics data (e.g. proteomics and metabolomics). The Horvath pan-tissue clock was designed to be used with all DNAm sources, excluding sperm, across the lifespan. It was developed using 7844 non-cancer samples from 82 DNAm datasets comprising 51 tissues and cell types [6]. However, the Horvath pan-tissue clock was found to perform poorly at estimating the age of fibroblasts (i.e. cells that create and maintain connective tissue) and other skin cells, so another clock was designed [7]. The Horvath skin & blood clock estimates DNAm-based age using 391 CpGs that strongly correlated with age in a variety of skin cells, blood, and saliva samples [7]. The Horvath skin & blood clock has also been shown to perform well in a variety of different cell and tissue types, including neuron and glial cells, brain samples, liver samples, and bone samples [7].

Following the development and widespread use of the Horvath clocks, clocks specific to perinatal and pediatric populations were developed. The Wu clock, which selected 111 age-related CpGs, was based on whole blood samples from 716 healthy children aged 9 to 212 months [8]. This clock was shown to estimate the biological age of child blood samples more accurately than previous clocks [8]. Pediatric epigenetic clocks were also developed for use with buccal cells. The Pediatric-Buccal-Epigenetic (PedBE) clock assessed DNAm profiles in buccal cell samples from 1721 individuals 0 to 20 years of age and selected 94 age-related CpGs [9]. Recently, the NEOage clocks were designed to accurately estimate postmenstrual and postnatal age (PNA) using buccal cells collected from 542 very preterm infants [10]. The NEOage PNA clocks were developed for two versions of the Illumina methylation platforms – the PNA clock for use with the 450K array (450K-PNA) selected 130 CpGs, whereas the clock for use with the EPIC array (EPIC-PNA) selected 318 CpGs. These clocks were found to predict age more accurately than the Horvath skin & blood and PedBE clocks in an external dataset of saliva samples from full and preterm infants [10].

#### Epigenetic clocks for gestational age prediction in perinatal samples

DNAm-based biomarkers have also been developed to predict the gestational age of two perinatal tissues, cord blood and placenta. Two research teams developed different gestational age estimators that were published in the same journal issue – the Knight clock [11] and Bohlin clock [12]. The Knight clock selected 148 CpGs based on cord blood DNAm data from 1342 neonates [11], while the Bohlin clock selected 96 CpGs^3^ based on an ultrasound-predicted gestational age model and cord blood DNAm profiles from 1753 neonates [12]. The following year, the Mayne clock was developed, which used 170 placental tissue samples from first trimester, second trimester, third trimester, and term pregnancies to predict gestational age based on 62 CpGs [13]. Subsequently, the Lee clock used 1289 placenta samples and selected 558 CpGs that predicted gestational age of placental tissue [14]. Lee and colleagues [14] reported that other existing epigenetic clocks, such as the Horvath pan-tissue and skin & blood clocks, as well as the Knight and Bohlin gestational age clocks, did not accurately predict gestational age of placental samples. Overall, the findings from the calibration of the different epigenetic clocks available for perinatal and pediatric tissues indicate that the precision and replicability of biological aging estimates and their associated phenotypes are shaped by tissue type [6,7,10,14] and sample population characteristics such as age [8–10].

### Early environmental chemical exposures and epigenetic clocks

A burgeoning body of work has begun to investigate the associations between early environmental exposures and epigenetic aging rates using samples from cohort and population-based studies. There are several classes of environmental pollutants, including trace elements, endocrine-disrupting chemicals, and air pollutants, that have been the focus of the environmental epigenetics work to date [16]. Although a comprehensive examination of epigenetic clock studies of prenatal and early childhood chemical exposures is beyond the scope of this review, a brief analysis of several interesting findings is provided to illustrate the value and challenges of using epigenetic clocks in this early developmental context. Furthermore, as there is variation in the epigenetic aging terminology and measures reported, the synopsis provided below and in Table 2 could assist readers in recognizing and interpreting common epigenetic aging terms and metrics.

**Table 2:** Summary of common epigenetic aging terms and metrics

Term	Definition	Calculation
**General terms**		
Biomarker of aging	Quantitative parameter(s) that predict biological age.	Derived from a biological material such as a tissue, fluid, and/or cell, either individually or to form a composite.
Epigenetic clock	A particular type of aging biomarker that predicts biological age based on patterns of DNA methylation at age-related CpGs on the genome.	The weighted average (formed by the regression coefficients) of the age-related CpGs selected by the penalized regression method [6].
Biological age	An individual’s estimated age based on the level of age-dependent biological changes (e.g. molecular changes such as DNA methylation).	Different biomarkers of aging measure this using specific parameters (e.g. telomere length, lymphocyte count, DNA methylation).
Chronological age	An individual’s age based on time since birth.	Time of birth subtracted from the time of interest^1^, expressed in units of time^2^.
**Epigenetic aging rate metrics**		
AMAR	Originally proposed by [17], a ratio of biological age to chronological age.	Biological age^3^ divided by chronological age.
EAAD	Difference between an individual’s biological age and chronological age.	Biological age^3^ minus chronological age.
EAA	Provides an estimate of whether individuals are aging faster or slower biologically than their chronological age.	The residuals obtained from regressing estimated biological age^3^ on chronological age. *Referred to as GAA when the residuals obtained from regressing gestational age^4^ on chronological age.
EEAA	Captures biological aging rate whilst incorporating age-related changes in blood cell composition. Commonly mentioned when using other clocks (e.g. PhenoAge, GrimAge).	The residuals obtained from regressing estimated biological age^3^ on chronological age not adjusted by blood cell counts.
IEAA	Captures biological aging rate independent of blood cell type proportions; by definition, this is not correlated with chronological age and is meant to capture cell-intrinsic properties of the aging process. Commonly measured using the Horvath pan-tissue clock (i.e. ‘HorvathAge’).	The residuals obtained from regressing estimated biological age^3^ on chronological age adjusted by blood cell counts.
**Accuracy metrics**		
Age correlation	The correlation between biological age and chronological age; it cannot be calculated in datasets where subjects have the same chronological age (e.g. cord blood samples from newborns).	Pearson correlation coefficient between biological age^3^ and chronological age.
(Median) Error	Can be used to assess inaccuracy of an epigenetic clock.	MAD between biological age^3^ and chronological age.
Average age acceleration	Can be used to determine whether the estimated biological age of a tissue is consistently higher or lower than expected.	Defined as the average difference between DNA methylation-based age and chronological age.

1 Time of interest defined by the research question (e.g., current date, time of disease state).

2 Common units of time for age are weeks, months, and years.

3 With respect to chronological age estimators, biological age refers to DNA methylation-based age.

4 With respect to gestational age estimators, this refers to gestational age as estimated based on DNA methylation patterns.

AMAR, apparent methylation aging rate. EAA, epigenic age acceleration. EAAD, epigenic age acceleration difference. EEAA, extrinsic epigenic age acceleration. GAA, gestational age acceleration. IEAA, intrinsic epigenic age acceleration. MAD, median absolute difference.

Lead is a non-essential trace element that is recognized as a developmental neurotoxicant. This heavy metal has received notable attention in environmental epigenetics, including as part of clock validation. Specifically, the Wu clock was evaluated using a dataset of 42 dry blood spots from individuals 3 months to 5 years of age; boys with blood lead levels >5 µg/dl had higher apparent methylation aging rates than those with blood lead below this level [8]. This finding is similar to results from a study that used a subsample of 521 participants from the Early Life Exposure in Mexico to Environmental Toxicants (ELEMENT) cohort. They found that prenatal and childhood lead exposure was associated with altered epigenetic age acceleration (EAA) in blood leukocytes in adolescents, particularly in males [18]. Interestingly, the results were time- and clock-specific; maternal first and second trimester blood lead levels were associated with increased PedBE EEA, maternal third trimester blood lead levels were associated with decreased Horvath skin & blood EAA, maternal tibia bone lead levels were associated with increased Horvath pan-tissue EAA, and no associations were found for the Wu clock [18]. These findings could suggest that epigenetic clocks may be differentially sensitive to the age-related epigenetic effects of toxic chemical exposures such as lead depending on the timing of the exposure and the biological sample used for DNAm profiling. However, it is also possible that technical variation or ‘epigenomic noise’ from the different DNAm arrays and/or clocks themselves may affect epigenetic aging estimates [19,20].

Recent evidence also indicates that epigenetic clocks are differentially sensitive to the effects of air pollution. A study of 76 South Korean maternal–child pairs from the Environment and Development of Children cohort provided a comprehensive examination of early life exposure to air pollution and EAA [21]. The researchers considered a range of air pollutants, including particulate matter (PM_2.5_, PM_10_), nitrogen dioxide (NO_2_), ozone (O_3_), carbon monoxide (CO), and sulfur dioxide (SO_2_) throughout pregnancy and for one year prior to follow-up when the children were age 6. EAA was estimated in whole blood at age 6 using the Horvath clocks, PedBE clock, and Wu clock. Results revealed that each interquartile range increase in PM_2.5_ during pregnancy and in the one year prior to follow-up was associated with increased Horvath pan-tissue EAA, while each interquartile range increase in CO during pregnancy was associated with increased EAA as measured by both Horvath clocks [21]. They also found that each interquartile increase in PM_2.5_ in the one year prior to follow-up was associated with increased Wu EAA, and each increase in PM_10_ and O_3_ was associated with increased EAA as measured by Horvath’s pan-tissue and skin & blood clock, respectively [21]. Taken together with the previously mentioned studies that examined early lead exposure, these findings underscore the importance of including multiple epigenetic clocks in environmental epigenetics research, when possible, as specific clocks may capture age-related epigenetic changes associated with some environmental exposures but not others.

Common endocrine-disrupting chemicals used in plastics, such as phthalates and bisphenols, have also been linked to altered epigenetic aging rates in perinatal and pediatric tissues. One recent study examined 385 maternal–child pairs from the Center for the Health Assessment of Mothers and Children of Salinas (CHAMACOS) cohort and DNAm in blood samples [22]. These samples include cord blood that was used to estimate gestational age acceleration (GAA) using Bohlin’s clock, and blood clot samples that were used to estimate intrinsic EAA (IEAA) using the Horvath pan-tissue clock. The researchers found that higher prenatal exposure to di(2-ethylhexyl) phthalate was associated with lower IEAA in males at age 7, and higher exposure to a mixture of phthalates was marginally associated with lower GAA in males at birth [22]. Another study used 224 maternal–infant pairs from the Canadian APrON pregnancy cohort to examine prenatal exposure to bisphenols and phthalates and EAA estimated in buccal epithelial cells from infants at 3 months of age using the PedBE clock [23]. Results revealed that higher prenatal exposure to bisphenol A was associated with increased EAA in female infants, while higher prenatal exposure to several phthalate metabolites [e.g. mono-methyl phthalate (MMP), mono-carboxy-isooctyl phthalate (MCOP)] was associated with decreased EAA in female infants [23]. Notably, 1000 or more environmental chemicals are identified as endocrine disruptors. These findings highlight that different chemicals within the same class (e.g. endocrine-disrupting chemicals) or within the same family but with different molecular features (e.g. different molecular weight phthalates) could exhibit different effect directions with respect to DNAm-based aging.

Under the umbrella of the ‘exposome’ (i.e. the totality of exposures throughout an individual’s lifetime) [24], some research has started to examine a wider variety of chemical exposures, including organochlorine compounds (OCs), organophosphate pesticides (OPs), polybrominated diphenyl ethers, and per- and polyfluoroalkyl substances. The Human Early-Life Exposome project, which is based on six ongoing longitudinal population-based birth cohorts from across Europe, examined the associations between 83 prenatal and 103 childhood exposures and Horvath skin & blood IEAA in a sample of 1173 children aged 7 [25]. They found that prenatal and childhood exposure to tobacco smoke was associated with increased IEAA, while childhood exposure to one OP (i.e. dimethyl dithiophosphate) and one OC (i.e. polychlorinated biphenyl-138) was associated with decreased IEAA; however, these associations did not survive the correction for multiple comparisons [25]. This study illustrates how broad environmental epigenetic studies of the ‘chemical exposome’ may face statistical challenges (e.g. power, multicollinearity, difficulty examining mixture effects).

In summary, there are opportunities for novel and groundbreaking research to be conducted at the intersection of environmental epigenetics and biological aging in early development. Based on the insights from previous research (Table 3), investigators in this area are encouraged to study how the direction and magnitude of associations may differ based on the level of exposure [8], timing of exposure [18,21], specific chemical and/or class of chemical considered [23], and sex [18,22,23]. It will also be helpful to keep apprised of newly developed biostatistical and machine learning tools that enable investigation of multiple classes of environmental contaminants, chemical mixture effects, and the chemical exposome.

**Table 3:** Summary the reviewed epigenetic clock studies of prenatal and early childhood chemical exposures

First author, year published	Chemical exposure(s)	Sample characteristics and DNA methylation platform	Epigenetic clock(s) and metric(s)	Main findings
Wu, 2019 [8]	Lead levels above or below 5 µg/dl quantified from 3 mm punch-outs of blood spots on filter paper from young children	Young children from Detroit, Michigan, U.S.A. Dataset of 42 dry blood spots from 24 boys and 18 girls aged 3 months to 5 years were profiled for DNA methylation on the Illumina 450K array.	Wu clock was used to estimate EAAD and AMAR	EAAD and AMAR were significantly higher in boys with blood lead levels >5 μg/dl than in boys with blood lead below this level (EAAD: *P*=0.01, AMAR: *P*=0.003)
Halabicky, 2024 [18]	Lead concentrations measured in maternal blood from each trimester of pregnancy and child blood at 12- and 24-months of age. Maternal patella and tibia bone lead (as measures of cumulative prenatal lead exposure).	Maternal–child pairs from a sub-sample of the ELEMENT study Blood leukocytes from between 264 and 390 adolescents (mean age=14.5 years) were profiled for DNA methylation on the Illumina EPIC array.	Horvath pan-tissue clock was used to estimate EAA; IEAA and EEAA were also estimated. The online new methylation age calculator was used to estimate EAA based on the Horvath skin & blood, Hannum, PhenoAge, and GrimAge clocks. The *methylclock* package in R was used to estimate EAA based on the PedBE and Wu clocks.	Maternal tibia bone lead was positively associated with Horvath pan-tissue EAA (95% CI: 0.001, 0.15). First trimester maternal blood lead was positively associated with Horvath pan-tissue EEAA (95% CI: 0.03, 0.25), but third trimester maternal blood lead was negatively associated with Horvath pan-tissue EEAA (95% CI: −0.15,–0.003). First and second trimester maternal blood lead levels were positively associated with PedBE EAA (95% CIs: 0.004, 0.03). Third trimester maternal blood lead (95% CI: −0.09,–0.02) and 24 month blood lead levels (95% CI: −0.09,–0.005) were negatively associated with Horvath skin & blood EAA. There were multiple significant associations in sex-stratified models; generally, males demonstrated ‘faster’ biological aging rates, compared with females who demonstrated ‘slower’ biological aging rates, with respect to the metrics estimated by the Horvath pan-tissue and PedBE clocks.
Lee, 2024 [21]	Air pollution, including particulate matter [<2.5 µm (PM_2.5_) and <10 µm (PM_10_)], NO_2_, O_3_, CO, and SO_2_ were estimated based on residential address for the whole of pregnancy and for 1 year before the follow-up at child age 6.	Maternal–child pairs from a sub-sample of the EDC study. Whole blood samples collected from 80 children around 6 years of age (±1 month) were profiled for DNA methylation on the Illumina 450K array.	The *methylclock* package in R was used to estimate EAA based on the Horvath pan-tissue, Horvath skin & blood, PedBE, and Wu clocks.	For exposure during the whole of pregnancy, exposure to PM_2.5_ (95% CI: 0.031, 0.782) and CO (95% CI: 0.362, 1.196) were positively associated with Horvath pan-tissue EAA. For exposure during the 1 year before children were age 6, exposure to PM_2.5_ was positively associated with Horvath pan-tissue (95% CI: 0.043, 0.975) and Wu EAA (95% CI: 0.030, 0.548). For exposure during the 1 year before children were age 6, exposure to PM_10_ and O^3^ were positively associated with Horvath pan-tissue (95% CI: 0.029, 0.530) and skin & blood EAA (95% CI: 0.006, 0.320), respectively
Khodasevich, 2023 [22]	Average concentrations of 11 individual phthalate metabolites and a summary measure of DEHP from maternal urine samples collected at 13- and 26-weeks of gestation.	Mother–child pairs from a sub-sample of the CHAMACOS study. Cord blood and child blood clot samples collected from between 179–311 participants were profiled for DNA methylation on the Illumina 450K (cord blood, child samples from age 9) and EPIC arrays (child samples from age 7 and 14).	Bohlin’s GAA was estimated using the *GAprediction* R package The online new methylation age calculator was used to estimate Horvath’s pan-tissue ‘DNAm age’ (i.e. DNA methylation-based age/biological age) and IEAA for the child blood clot samples.	Maternal average mono-benzyl phthalate (MBzP) exposure was negatively associated with GAA at birth (95% CI: −0.947 to −0.019). Maternal average DEHP exposure was negatively associated with IEAA among males at age 7 (95% CI: −1.06,–0.18)
England-Mason, 2024 [23]	Concentrations of 12 phthalate metabolites, 3 summary measures of phthalates (i.e. molar sums of DEHP and high and low molecular weight phthalates), and 2 bisphenols from maternal urine samples collected during the second trimester (mean=17 gestational weeks).	Mother–child pairs from a sub-sample of the APrON study. BEC swabs collected from 224 infants (mean age=13.1 weeks) were profiled for DNA methylation on the Illumina EPIC array.	The publicly available script was used to estimate PedBE EAA. The online new methylation age calculator was used to estimate Horvath’s pan-tissue EAA.	Maternal second trimester bisphenol A (BPA) concentrations were positively associated with PedBE EAA in all infants (95% CI: 0.29, 1.03). Several maternal second trimester phthalate metabolite [MMP, MCOP, mono-isononyl phthalate (MNP)] concentrations were negatively associated with PedBE EAA in female infants (95% CIs: −1.28,–0.02), and MNP was negatively associated with Horvath pan-tissue EAA in female infants (95% CI: −0.07,–0.01)
de Prado-Bert, 2021 [25]	A broad range of environmental exposures (i.e. urban exposome and air pollution exposures; maternal and child blood and urine samples were collected to assess OCs, OP metabolites, PBDEs, PFAS, essential minerals, non-essential minerals, phenols, phthalate metabolites, and cotinine; water disinfection by-products; other lifestyle factors such as diet and physical activity), amounting to 83 prenatal and 103 childhood exposures.	Maternal–child pairs from a sub-sample of the HELIX study. Buffy coat blood samples collected from 1173 children (mean age=8.1 years) were profiled for DNA methylation on the Illumina 450K array.	The *methylclock* package in R was used to estimate ‘DNAm age’ (i.e. DNA methylation-based age/biological age), ‘ageAcc’ (i.e. EAAD), ‘ageAcc2’ (i.e., EAA), and EAA adjusted for cell-type proportion (i.e. IEAA) based on the Horvath pan-tissue, Horvath skin & blood, PedBE, and Wu clocks. The online new Methylation Age Calculator was also used to estimate Horvath’s pan-tissue epigenetic aging rate metrics.	The Horvath skin & blood clock showed the strongest correlation with chronological age (*R*=0.85, *P*<0.001) in the study sample, so Horvath skin & blood IEAA was used as the main outcome. For the prenatal exposome, maternal tobacco smoke during pregnancy was positively associated with Horvath skin & blood IEAA (95% CI: 0.02, 0.26). For the childhood exposome, indoor particulate matter absorbance (PM_abs_; 95% CI: 0.02, 0.12) and parental smoking (neither vs. both parents; 95% CI: 0.01, 0.29) were positively associated with Horvath skin & blood IEAA. For the childhood exposome, the OP dimethyl dithiophosphate (DMDTP) (undetected vs. detected; 95% CI: −0.24,–0.02) and polychlorinated biphenyl-138 (PCB-138; 95% CI: −0.14,–0.01) were negatively associated with Horvath skin & blood IEAA.

AMAR, apparent methylation aging rate. APrON, Alberta Pregnancy Outcomes and Nutrition. BEC, buccal epithelial cell. BPA, bisphenol A. CHAMACOS, Center for the Health Assessment of Mothers and Children of Salinas. CO, carbon monoxide. DEHP, di (2-ethylhexyl) phthalate. DMDTP, dimethyl dithiophosphate. EAA, epigenic age acceleration. EAAD, epigenic age acceleration difference. EDC, Environment and Development of Children. EEAA, extrinsic epigenic age acceleration. ELEMENT, Early Life Exposure in Mexico to Environmental Toxicants. HELIX, Human Early-Life Exposome. IEAA, intrinsic epigenic age acceleration. MBzP, mono-benzyl phthalate. MCOP, mono-carboxy-isooctyl phthalate. MMP, mono-methyl phthalate. NO_2_, nitrogen dioxide. O_3_, ozone. OCs, organochlorine compounds. OPs, organophosphate pesticides. PBDEs, polybrominated diphenyl ethers. PFASs, per- and polyfluoroalkyl substances. SO_2_, sulfur dioxide.

### Considerations for using epigenetic clocks in environmental epigenetics

When selecting the best epigenetic clock to address a research question, researchers should consider: (i) tissue type, (ii) cell type heterogeneity, and (iii) relevance to exposure and/or phenotype of interest. Tissue type is a critical consideration and may be largely determined based on study design and feasibility, as it is challenging to collect invasive biological samples, such as blood, in young children and in non-clinical settings. Conversely, saliva or buccal samples can be easier to obtain. However, these matrices have very different cell type compositions. For example, buccal samples are primarily composed of a single cell type (i.e. buccal epithelial cells), while blood is heterogeneous and different blood samples have distinctive cell populations and proportions (e.g. all blood cell types in whole blood, mostly lymphocytes in peripheral blood mononuclear cells, nucleated red blood cells and other cell types in cord blood). Researchers should use reference-based methods (e.g. [26]) when available or deconvolution methods to estimate cell type composition and account for this in statistical analyses. Related to these first two considerations, it is also important to consider which tissue is most pertinent to the exposure and/or phenotype of interest. For example, buccal cells may be more informative than blood samples in epigenetic research investigating non-blood-based diseases and phenotypes, given the shared ectodermic origin of buccal and brain tissue [27]. Notably, some epigenetic clocks were developed for use with specific tissue and/or cell types (e.g. Knight and Bohlin clocks for cord blood, PedBE for buccal cells), while others are more broadly applicable (e.g. Horvath pan-tissue clock). Table 4 presents a summary of relevant information to aid in the selection of the best epigenetic clock based on tissue and/or cell type(s), population to be studied, and accessibility.

**Table 4:** Recommendations and considerations for use of the epigenetic clocks

Clock, year published	Tissue type(s) used for clock validation	Populations recommended for use	Recommended tissues	Not recommended tissues	Accessibility^2^
**Chronological age estimators**					
Horvath pan-tissue, 2013 [6]	51 Tissues and cell types (see [6] for further detail)	Neonates, infants, children, adolescents, adults, and elderly adults	Recommended for heterogenous tissues (e.g. whole blood, PBMCs, cerebellar samples, occipital cortex, buccal epithelium, colon, adipose, liver, lung, saliva, and uterine cervix) and individual cell types (e.g. CD4 T cells and CD14 monocytes).	Not recommended for breast tissue, uterine endometrium, sperm, dermal fibroblasts, skeletal muscle tissue, and heart tissue.	R software tutorial and annotation files available as supplementary materials [6]. Publicly available at: https://dnamage.clockfoundation.org/
Horvath skin & blood, 2018 [7]	Fibroblasts, keratinocytes, buccal cells, endothelial cells, blood, and saliva	Neonates, infants, children, adolescents, adults, and elderly adults	Recommended for various somatic cell types (e.g. fibroblasts, keratinocytes, and endothelial cells), peripheral blood samples, and skin biopsies.	Other tissue types	R software code available as supplementary materials [7]
Wu, 2019 [8]	Whole blood	Infants, children, and adolescents	Recommended for pediatric blood samples.	Other tissue types	Computed as stated in [8] The regression coefficients are available in the supplementary materials and the F function is defined in the article
PedBE, 2020 [9]	Buccal cells	Infants^3^, children, adolescents, and young adults (>20 years)	Recommended for pediatric buccal cells. Could be considered for pediatric saliva or blood samples, as well as for adult buccal samples^1^.	Other tissue types	R software code is publicly available online: https://github.com/kobor-lab/Public-Scripts/
NEOage, 2021 [10]	Buccal cells	Preterm infants (<30 weeks’ gestation) Could be considered for full-term infants^3^	Buccal cells or saliva from preterm infants. Could be considered for buccal cells or saliva from full-term infants^3^.	Other tissue types	R software code and the corresponding coefficients are available as supplementary materials [10]
**Gestational age estimators**					
Knight, 2016 [11]	Cord blood	Neonates	Cord blood	Other tissue types	R software code and documentation are available as supplementary materials [11]
Bohlin, 2016 [12]	Cord blood	Neonates	Cord blood	Other tissue types	The corresponding coefficients for the different models of gestational age (e.g. ultrasound-predicted) are available as supplementary materials [12]
Mayne, 2017 [13]	Placenta	First, second, and third trimester, and term pregnancies	Placenta	Other tissue types	The corresponding coefficients are available in the supplementary materials [13]
Lee, 2019 [14]	Placenta	First, second, and third trimester, and term pregnancies	Placenta	Other tissue types	The corresponding coefficients are available as supplementary materials [14]

1 The PedBE clock demonstrates moderate performance in pediatric saliva (r=0.50, error=1.31 years) and pediatric blood samples (r=0.79, error=3.26 years; when adjusting for blood cell type variance) as well as adult buccal samples (r=0.72, error=1.40 years) [10].

2 R packages are publicly available that estimate chronological and gestational age. For example, *‘methylclock’* is available from Bioconductor at https://www.bioconductor.org/packages/release/bioc/html/methylclock.html and estimates Horvath’s pan-tissue and skin & block clocks, the PedBE clock, Wu’s clock, Knight’s clock, Bohlin’s clock, Mayne’s clock, and Lee’s clock.

3 For more information regarding the use of the PedBE and/or NEOage clocks for samples of full-term infants, see the ‘Considerations for Using Epigenetic Clocks in Environmental Epigenetics’ section of this review, as well as [28] and [11].

In addition to the above recommendations for thoughtfully selecting the most appropriate epigenetic clock for addressing the primary research question, researchers are also encouraged to consider how their clock selection compares to the overall body of literature. Most of the studies examining epigenetic clocks in perinatal and pediatric populations have used blood samples (e.g. cord blood, whole blood, and dry blood spots), but some studies have used saliva or buccal cell samples [29]. Furthermore, most studies have used the Horvath pan-tissue clock or multiple clocks [29]. Thus, it is also suggested that researchers conduct supplementary analyses that examine other relevant clocks to improve interpretation and consistency across studies. Determining the most appropriate epigenetic clock or clocks for use in longitudinal research or multi-cohort designs may be additionally complex due to methodological differences (e.g. tissues available and timepoints of sample collection), but these same considerations can still be applied.

Another important consideration for future environmental epigenetic studies that use epigenetic clocks is that the specific platform and/or version used to generate DNAm data can affect the availability and measurement of CpGs used to derive epigenetic aging metrics. There has been limited investigation of the stability of DNAm measurements across the evolution of the Illumina methylation platforms, and technical variability may present issues for epigenetic clock studies. Although the transition from the Illumina 450K array to the EPIC array was shown to result in relatively stable EAA estimates [30], this may not be the case for comparisons across the three most recent generations of the Illumina arrays. For example, the variability in missingness of impactful CpGs on different Illumina (i.e. 450K, EPIC, and EPIC v.2) arrays^4^ has been shown to result in discrepant epigenetic age estimates, and it has been suggested that future studies should consider using a principal component analysis approach to derive EAA to enhance reliability [20]. A tool that may be helpful in evaluating the consistency of measurements across the Illumina 450K, EPIC, and EPIC v.2 arrays is the Cross-Array Comparison and Testing Interface (https://cacti.geddes.rcac.purdue.edu/). However, the technical variation across arrays (e.g. number of probes, differences in probe naming conventions, genomic distribution of probes, and novel inclusion of replicate probes on the EPIC v.2 array) presents challenges for bioinformatics analyses and interpretations of DNAm data generated by different arrays within and across studies; although the newest version (EPIC v.2) is considered a ‘worthy successor’ [31]. The technical differences across arrays will be especially important to account for in longitudinal studies and meta-analyses, and may present challenges for harmonizing data, although the most robust and recommended approach is to calculate EAA (the ‘regression-based measure’) separately for samples profiled on different versions of the array [28].

A recent evaluation of chronological and gestational age estimators based on data from seven tissue types and three DNAm arrays provides guidance for the future use of epigenetic clocks in perinatal and pediatric samples [32]. Specifically, Fang and colleagues [32] recommended the Lee clock for use with placenta, the Bohlin and Knight clocks for use with neonatal cord blood (i.e. Bohlin clock has a higher correlation with gestational age, but the Knight clock had smaller median errors), the Horvath pan-tissue clock for children’s blood samples, and the PedBE clock for children’s buccal samples. The NEOage clock is recommended for buccal samples from preterm infants [10] and can also be used for buccal samples from full-term infants [10,32], but more research is needed to determine the respective performance of the NEOage and PedBE clocks in very young infants. The Fang et al. [32] analysis did not examine all readily available epigenetic clocks (e.g. Wu clock), but based on previous research [7,8,10,13,21,33], additional recommendations for the other previously described clocks are made in Table 4.

### Future directions

Epigenetic markers can offer novel insights into the biological pathways connecting environmental exposures to health, but it is important to note that epigenetic clocks are an example of an informative, but non-causal biomarker of exposure and disease risk. As such, it is appropriate to examine epigenetic clocks in relation to environmental chemical exposures, health and disease outcomes, and as biological mediators, but they are not etiological agents. Unfortunately, the reliability of some epigenetic aging biomarkers and the cost of DNAm profiling hinders their current clinical use, but future research may help validate them as surrogate endpoints for regulatory purposes, determine which exposures or interventions decrease or increase the healthspan, and identify individuals who may benefit from treatment [34]. Relatedly, much research to date has focused on the ‘first-generation epigenetic clocks’ that predict chronological age (e.g. Horvath pan-tissue clock), while second-generation clocks were designed to predict phenotypic measures (e.g. GrimAge predicts mortality risk), and one next-generation clock even captured the pace of aging (e.g. DunedinPACE/DunedinPoAm algorithm) [35]. Moving forward, environmental epigenetics researchers should consider developing and training epigenetic clocks using pediatric clinical and developmental indicators. This approach may help clarify age-related mechanisms linking environmental chemical exposures to adverse child health and developmental trajectories.

Presently, more basic research is needed as the relationship between epigenetic aging rates and health in early development is likely distinctive from adulthood [2,8]. In younger populations, it has been proposed that EAA may indicate greater developmental maturity [2]. It has also been suggested that whether increased or decreased, some deviations in EAA may indicate adverse growth and physical development, while others may confer risk for neurodevelopmental difficulties and future psychopathology [23]. The application of epigenetic clocks in a pediatric context can differentiate individuals with positive or increased EAA (those aging ‘faster’) from those with negative or decreased EAA (those aging ‘slower’), but what this means may not be as simplistic as increased EAA being indicative of early ‘aging’ or greater maturity [33]. It is more likely that epigenetic age deviations reflect epigenetic programming by early life environmental exposures which affect child outcomes and development [23,33], but the interpretation based on effect direction is challenging and still up for debate. Unraveling these nuances will be complex, as the influence of prenatal and early childhood chemical exposures on epigenetic aging rates may only manifest during certain periods of childhood [22], or in certain tissues, or be detectable by certain epigenetic clocks.

A recent systematic review highlighted a variety of biological, social (e.g. sociodemographic factors such as race and socio-economic disadvantage), and environmental factors that have been consistently associated with EAA [36]. For example, across a range of age groups and epigenetic clocks, male sex has been robustly associated with increased EAA [36]. It is possible that this sex difference may be consistent with the ‘male–female health-survival paradox’ (i.e. females experience greater longevity but higher rates of poorer health outcomes and disability than males) [37]. It has also been suggested that this male bias toward faster biological aging could reflect a higher baseline of DNAm-based age, greater rates of change, and/or greater age-associated genomic instability [36]. It is difficult to speculate further given the limited investigation of potential sex differences in epigenetic age estimates in newborns and young children, but some gestational age estimators (e.g. Knight and Bohlin clocks) show sex differences in clock accuracy metrics (e.g. age correlation and median error) [32]. Strong evidence of sex differences in DNAm profiles in early life comes from a meta-analysis of 17 cohorts participating in the Pregnancy And Childhood Epigenetics (PACE) consortium. This meta-analysis revealed small but consistent sex differences in neonatal and childhood DNAm levels at >30,000 CpGs, and these were enriched in genes involved in pathways related to neurodevelopment, mental health, and cardiovascular health [38]. To help provide fundamental information on the health and developmental implications of epigenetic age deviations in perinatal and pediatric populations, it is imperative that environmental epidemiologists and epigeneticists collaborate to develop and validate epigenetic clocks specifically for perinatal and pediatric populations, including training and testing these clocks using multiple tissue types, multiple methylation platforms, different sample populations (e.g. based on race, socioeconomic status, sex), and in relation to different measures of development.

## Conclusion

Environmental epigenetics is a field in relative infancy, but it offers many opportunities for understanding if environmental chemical exposures make epigenetic clocks tick. Future research is needed to better understand the functional implications of epigenetic modifications accumulated during the aging process and how these establish the aging phenotype [39]. This is especially true for the prenatal and early childhood periods. Epigenetic biomarkers hold promise for the early detection and prediction of disease, and it is possible that aging biomarkers such as epigenetic clocks may be useful markers that reflect epigenotoxicity.

**Summary Points**

- Epigenetic clocks are a type of aging biomarker that can offer insight into age-related changes associated with early environmental exposures.
- Epigenetic clocks are devised using a supervised machine learning method to estimate DNA methylation-based aging in human tissues.
- Research that has examined associations between early environmental chemical exposures and epigenetic aging rates has focused on certain exposures, such as endocrine-disrupting chemicals.
- When selecting the best epigenetic clock to address a research question, researchers should consider tissue type, cell type heterogeneity, and relevance to the exposure and/or phenotype of interest.
- More research is needed that calibrates clocks specifically for perinatal and pediatric populations to better understand the implications of early epigenetic age deviations.

## Footnotes

1 Elastic net regression is a combination of LASSO (L1) and ridge (L2) regularization methods, that balances two tuning parameters (α and λ) to emphasize model sparsity and consider correlated variables.

2 The first human methylation array, the Illumina Infinium Human Methylation27 (27K), was released in 2008 and measured methylation levels at over 27,000 CpGs. This was followed in 2011 by the release of the Illuminia Infinium Human Methylation450 (450K) array, which targeted over 450,000 methylation sites; the most recent version of the Illumina Infinium MethylationEPIC (EPIC) array has increased coverage of the epigenome (i.e., >850,000 CpGs on the 2016 EPIC or 850K, >900,000 CpG sites on 2023 EPIC v2.0 or 900K).

3 The other models selected 58–132 CpGs and were based on other gestational age estimates (i.e., last menstrual period, ultrasound and Bonferroni adjusted, last menstrual period and Bonferroni adjusted, ultrasound and false discovery rate adjusted, last menstrual period and false discovery rate adulted).

4 For example, the CpGs included in the Horvath pan-tissue clock are all covered (0% missing) on the 450K, but 19% are missing on the EPIC and 13% missing on the EPIC v.2; while the CpGs included in the Horvath skin & blood clock are fully covered (0% missing) on both the 450K and EPIC arrays, but 17% missing on the EPIC v.2.

## Open Access

This article has been published open access under our Subscribe to Open programme, made possible through the support of our subscribing institutions, learn more here: https://portlandpress.com/pages/open_access_options_and_prices#conditional

## Abbreviations

CO: carbon monoxide
CpG: cytosine-phosphate-guanine (a specific sequence of DNA nucleotides)
DNAm: DNA methylation
EAA: epigenetic age acceleration
EDC: Environmental and Development of Children
EPIC: illumina infinium methylationEPIC BeadChip
GAA: gestational age acceleration
IEAA: intrinsic epigenetic age acceleration
450K: illumina infinium HumanMethylation450 BeadChip
O_3_: ozone
OC: organochlorine compound
OP: organophosphate pesticide
PM_10_: particulate matter (particles that are 10 microns or less in diameter)
PM_2.5_: fine particulate matter (particles that are 2.5 microns or less in diameter)
PNA: postnatal age
PedBE clock: Pediatric-Buccal-Epigenetic clock
